# A 22-Site Comparison of Land-Use Practices, *E-coli* and Enterococci Concentrations

**DOI:** 10.3390/ijerph192113907

**Published:** 2022-10-26

**Authors:** Jason A. Hubbart, Elliott Kellner, Fritz Petersen

**Affiliations:** 1 Division of Forestry and Natural Resources, Davis College of Agriculture, Natural Resources and Design, West Virginia University, Percival Hall, Morgantown, WV 26506, USA; 2 The Donald Danforth Plant Science Center, 975 N. Warson Rd, St. Louis, MO 63132, USA; 3 Department of Biology, Biology Life Sciences Building, Loyola University Chicago, 1032 W. Sheridan Road, Chicago, IL 60660, USA

**Keywords:** *Escherichia coli*, enterococcus, fecal indicators, land use practices, acid-mine drainage, water quality

## Abstract

Land-use practices can greatly impact water quality. *Escherichia (E.) coli* and Enterococcus are accepted water quality indicators. However, surprisingly little research has been conducted comparing both organisms’ population density relationships to land use practices and water quality. Stream water grab samples were collected monthly (*n* = 9 months) from 22 stream monitoring sites draining varying land use practice types in a representative mixed-land-use watershed of the northeastern United States. *E. coli* and enterococci colony forming units (CFU per 100 mL) were estimated (*n* = 396) and statistically analyzed relative to land use practices, hydroclimate, and pH, using a suite of methods, including correlation analysis, Principal Components Analysis (PCA), and Canonical Correspondence Analysis (CCA). Correlation analyses indicated significant (*p* < 0.05) relationships between fecal indicator bacteria concentrations, water quality metrics and land use practices but emphasized significant (*p* < 0.05) negative correlations between pH and instream enterococci concentrations. PCA and CCA results indicated consistent spatial differences between fecal indicator bacteria concentrations, pH, and land use/land cover characteristics. The study showed that pH could be considered an integrated proxy variable for past (legacy) and present land use practice influences. Results also bring to question the comparability of *E-coli* and enterococci relative to dominant land use practices and variations in pH and provide useful information that will help guide land use practice and water pollutant mitigation decision making.

## 1. Introduction

Aquatic fecal pollution results in unsafe water quality for many beneficial uses and accounts for approximately 10% of the annual global disease burden, including approximately 1.4 million child deaths each year. This alarming figure exceeds the combined rates of mortality from measles, malaria, and acquired immunodeficiency syndrome [1,2]. Freshwater fecal pollution and associated pathogenic bacteria, including (but not limited to) *Escherichia (E) coli*, and enterococci can result in urinary tract infections, diarrhea, and various respiratory illness [3,4]. For land managers and policy makers to improve land-use management decisions, reduce outbreaks of waterborne disease, and improve public safety in surface waters, improved understanding of factors affecting fecal concentrations in environmental waters is needed. However, many hydrological and geochemical relationships relative to microbial population dynamics remain poorly understood. For example, recent investigations indicated that the occurrence and environmental persistence of fecal microbes may be altered by the physicochemical condition of receiving water in which they occur [5,6,7]. Thus, investigations are greatly needed that will improve understanding of factors affecting the presence and concentrations of fecal pollution.

*E. coli* are anaerobic, Gram-negative, gamma-proteobacteria [8,9]. They are commonly found in the intestines of humans and other endotherms [10]. *E. coli* are most often commensal with the host organism and therefore, most often, cause no harm [11]. However, some *E. coli* strains are known to cause disease in humans and other endotherms [8,11]. Even non-pathogenic strains can lead to infection in individuals with weakened immune systems [12,13]. Additionally, given that *E. coli* can transmit cross-species, outbreaks can include large portions of populations, and may result in outbreak epidemics [12]. Enterococci (formerly known as the fecal or Lancefield group D streptococci) are spherical or ovoid-shaped (cocci). They are facultatively anaerobic, Gram-positive, and non-spore-forming that occur singly, in pairs, or in short chains [5,14,15,16]. Sources of enterococci can range from environmental (soil and water) to animal, bird, insect, and human sources, with the gastrointestinal tracts of animals serving as the primary habitat [16,17]. Under favorable conditions, enterococci can propagate on the surfaces of plants [16,18]. Enterococci are most often commensal bacteria and are known to aid in digestion and related metabolic pathways. However, certain species, including *Enterococcus (E.) faecalis* and *E. faecium* are nosocomial etiological microbes that can cause urinary tract infections, endocarditis, neonatal infections, abdominal and pelvic infections, and several other conditions in humans. 

*E. coli* and enterococci are frequently used as interchangeable fecal indicator organisms, particularly for water quality analyses, given their common distribution by means of feces of animals [5,10,19]. Consequently, increases in fecal matter within a water source are conventionally assumed to yield similarly linear increases in concentrations of both microbes [20]. For example, previous research showed that land use practices that include agricultural practices are quite often associated with observed increased population densities (i.e., concentrations) of *E. coli* and enterococci relative to other land use practices [7,21]. These relationships are often attributed to the presence of livestock, where livestock population density has been shown to be positively correlated to higher fecal indicator organism concentrations [22]. Similarly, the application of manure fertilizers on agricultural lands has also been shown to increase fecal bacteria concentrations in fresh water supplies [7,21,23]. Thus, different land use practices, including agricultural animal (ruminant) management practices, can result in comparative disequilibrium of *E. coli* and enterococci concentrations in fresh water.

In their classic work, Ref. [24] showed that concentration differences of *E. coli* and enterococci were not only attributable to presence of animal feces, but also attributable to additional factors including fecal distribution and reproductivity in the environment, survivability in temperature and chemical treatments, and bacteria longevity. Importantly, early work, while highly informative, seldom included investigations that advanced understanding of population density related perturbations for both microbes, in tandem relative to land use practices. In soils, independent studies showed that enterococci populations rapidly decreased over a one-month period following simulated rainfall events [25]. Conversely, *E. coli* populations showed initial growth of four to 25 times the original inoculant concentrations in rainfall treated soil depths of 30 and 90 mm, respectively [25]. Other investigations showed prolonged environmental persistence of enterococci following expulsion into the environment [26,27,28]. For example, enterococci concentrations on drift seaweed at recreational beaches in New Zealand exceeded seawater enterococci concentrations by 2.4 orders of magnitude [26]. This observation was attributed to population growth occurring due to favorable conditions created by the seaweed. Additionally, high enterococci cell densities in soils were attributed to enhanced survival of Gram-positive bacteria (e.g., enterococci) compared to Gram-negative bacteria (e.g., *E. coli*) when subject to environmental stressors [5], particularly desiccation or cellular injury [29,30]. Ultimately, the extended non-enteric survival of both *E. coli* and enterococci has called into question their use as fecal indicators, given that short term environmental survival is one of the key requirements for accurate fecal indicator organisms [10,20,31]. Additionally, potentially contrasting environmental survival of these two organisms under similar conditions [24,32] raises question as to their equivalent efficacy as water quality bioindicators.

Previous investigators used many different study designs to improve the understanding of fecal coliform regimes. Methods range from laboratory-based studies [33] field-based studies [33], periodic sampling [34], stochastic sampling [35] and nested-scale experimental watershed designs [7,21,36,37,38,39]. Implementation of the experimental watershed study design has been shown successful for quantification of land use practice impacts on receiving waters in mixed land use watersheds [36,40,41,42,43,44,45,46]. Using this design, a larger watershed can be divided into smaller sub-catchments enabling investigation of land use practice influences on environmental variables [21,47,48]. This is important because in so-doing, hydrology and management practices may be isolated using sub-catchment delineations and control and treatment effects [36]. The design is successful because the method facilitates identification of influences and cumulative effects of a range of specific to multiple management practices on hydrology and water quality variable(s) of interest [49]. In context of the current work, the approach allows for identification of factors (e.g., land use impacts) impacting the response variable(s) of interest (e.g., concentration of fecal bacteria) [36].

The Appalachian region of the United States shares many environmental and water quality related challenges with various locations globally, including fecal pollution [50]. In addition, Appalachia has diverse physiography that includes broadly divergent ecological, geographic, and climate characteristics [51]. The year-round rainfall regime and temperate climate of Central Appalachia [52] is similar to climates of regions such as Southern Brazil or Uruguay [53]. Further, research that advances understanding of and therefore mitigation of problematic fecal pollution processes is needed that yields transferable methods and results to other regions. Ultimately, an increasing number of households have water quality issues, including microbial contamination, that impacts security of drinking water supplies, and freshwater recreation. Therefore, water quality and security constitute a primary concern globally [54]. Given these needs, the current investigation was undertaken to (a) quantitatively compare concentrations of *E. coli*, enterococci, and pH in freshwater streams (b) draining catchments of varying dominant land use practices (c) using a 22-site experimental watershed study design in a municipal mixed-land use watershed of Appalachia, USA. Study outcomes advance understanding of the impact of dominant land use practices (both historic and contemporary) on fecal contamination and in-tandem concentrations of *E. coli* and enterococci, thereby improving land-manager decision making ability in similarly complex mixed-land use watersheds.

## 2. Materials and Methods

### 2.1. Study Site Description

This research took place in West Run Watershed (WRW), a 23 km^2^ mixed-land use watershed located in the Appalachian Mountains in north central West Virginia, USA. Annual climate in West Virginia includes lack of dry season, cold winters (mean monthly temperature < 0 °C) and warm-to-hot summers (mean monthly temperature > 22 °C) [55]. On average, Morgantown receives approximately 1060 mm of precipitation annually (climate record 1981–2010), with the coldest (January) and driest (February) months having average daily temperature of −0.4 °C and an average monthly precipitation of 66 mm, respectively [56]. July is the warmest and wettest month with average daily temperature of approximately 23 °C and average monthly precipitation of 117 mm [56,57,58,59].

**Figure 1:**
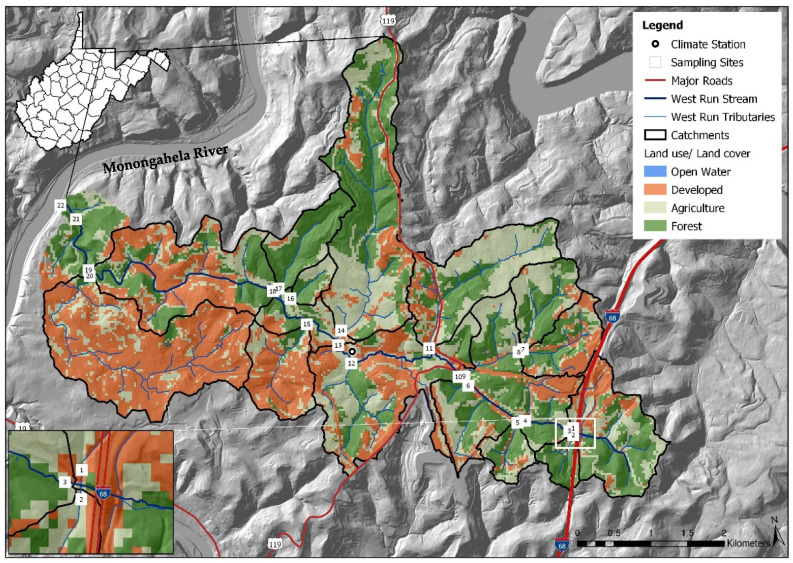
Monitoring site locations (*n* = 22) and land use cover, in West Run Watershed, Morgantown (Latitude: 39.629524, Longitude: −79.955894, WV, USA).

West Run Creek, the primary drainage of WRW, is a third order tributary of the Monongahela River [7]. For the current work, a 1/3 arc-second DEM was downloaded from the USGS National Elevation Dataset (https://www.usgs.gov, accessed on 15 February 2020) and land use data were downloaded from the National Land Cover Dataset (NLCD) and 2018 National Agriculture Imagery Program (NAIP to quantify land use types and develop Figure 1. WRW includes 42.7% forested, 37.7% mixed development (urban and commercial areas) and 19.4% agricultural land use practices. There are many other less spatially dominant, though arguably more environmentally impactful land uses, including historic mining, industrial, open water, and others. West Run Creek is a narrow, moderately entrenched stream with multiple small floodplains [21,60,61]. The elevation of the headwaters of WRW is 420 m above mean sea level [6,7], while elevation of the confluence with the Monongahela River is 240 m above mean sea level [21]. The watershed includes relatively rugged terrain, featuring numerous Paleozoic era rock outcroppings [7,21,61], more recent geological formation in the headwaters (Monongahela series), and two coal formations including the Upper Kittanning and the Pittsburg coal seams. Historic mining of the Pittsburg coal seam continues to negatively impact water quality in WRW (e.g., acid mine drainage), particularly in the headwaters [6,61].

A nested-scale and paired experimental watershed study design [36,62,63], including 22 study sites (i.e., gauge sites), was implemented in WRW in 2016 (see [6] and citations therein). Sampling sites (numbered in downstream order) were located on West Run Creek (#3, #4, #6, #10, #13, #18, #19, #21 and #22) and its first and second order confluence tributaries (#1, #2, #5, #7, #8, #9, # 11, #12, #14, #15, #16, #17 and #20), with contributing sub-catchments characterized by varying land use practices (Table 1; Figure 1). Study sites were identified using field survey data and GIS. Forested land use was the dominant land use type in all sub-catchments except those associated with monitoring sites #1, #11, #15, #16 and #20. Sub-catchments #1, #15 and #20 were primarily mixed development, whereas sub-catchments #11 and #16 were primarily agricultural (Table 1). Monitoring site #17, with 85.8% forested, 9.4% agricultural, and 4.8% mixed development land use practices served as a reference site (control). 

### 2.2. Data Collection

For the current study (3 March–3 November 2020) a Solinst Levelogger Gold pressure transducer was installed at site #13 (central watershed) to monitor stream flow. The sensor logged stream water stage (water depth, cm), with accuracy ± 0.3 cm at 30-min intervals (Figure 1). Precipitation was recorded with a Campbell Scientific TE525 Tipping Bucket Rain Gauge, and air temperature (Campbell Scientific HC2S3 Temperature Probe) was recorded at 3 m height at a climate station located within approximately 30 m of Site #13 (Figure 1). For the current work, monthly stream water grab-samples were collected as per the methods of [6,48,64] from each monitoring site (stream order ≤ 3). Water samples were collected on the first Tuesday of each month. At the same time as water sampling, a YSI Pro-Series DSS handheld water quality sonde was used to collect pH values at each sampling site. The sensor-bearing sonde tip was inserted into the stream to approximately 60% depth, to describe a representative sample as per methods detailed in [65]. The YSI was calibrated before every use, as per manufacturer instructions (YSI, Inc., Yellow Springs, OH, USA). The sampling period for the study was approximately eight months (3 March–3 November 2020), which spanned the growing season, and consisted of nine sampling events for each of the 22 monitoring sites (Table 1, Figure 1). The study design resulted in large sample size of 396 (nine monthly samples from 22 sites and two distinct microbe tests) spatiotemporally distinct *E. coli* and enterococci concentration values.

Following collection, stream water samples were transported to the Interdisciplinary Hydrology Laboratory, located in the Davis College of Agriculture, Natural Resources and Design at West Virginia University, for analyses. In the laboratory, fecal contamination was quantified immediately upon arrival at the laboratory using *E. coli* and enterococci as indicator organisms [6,10,21]. *E. coli* and enterococci colony forming units (CFU) were quantified using the U.S. Environmental Protection Agency (EPA) approved Colilert and Enterolert tests, respectively [66], developed by IDEXX Laboratories Inc. (Westbrook, Maine, USA) The tests utilize a most probable number (MPN) approach to estimate the *E. coli* and enterococci CFU concentration. Both tests, included in Standard Methods for Examination of Water and Wastewater, were developed to estimate fecal concentrations in water samples without requiring sample dilution [66,67]. As per the method instructions [66], the Colilert test’s (*E. coli* test) combination of Colilert’s Defined Substrate Technology nutrient-indicators (ortho-Nitrophenyl-β-galactoside (ONPG) and 4-methylumbeilliferyl-beta-D-glucuronide (MUG)) and a selectively suppressing formulated matrix create low chances of recording inaccurate results (likelihood of false positives ±10%). With this test, most non-target organisms are unable to grow, given that they lack the enzyme to metabolize the provided carbon source (ONPG and MUG) [66]. The formulated matrix selectively suppresses the few non-target organisms that can metabolize ONPG [66]. The method requires 100 mL of sampled water and uses a Quanti-Tray system, comprising 97 total wells (48 large wells, 48 small wells, and one overflow well). The Colilert substrate (ONPG and MUG) was added to 100 mL of sampled water, sealed in the Quanti-Tray, and incubated at 35 °C for 24 h, as per manufacturer instructions [66]. During the Enterolert test (enterococci test), 4-methyl-umbelliferyl ß-D-glucoside replaced ONPG and MUG as a nutrient indicator. The Enterolert (4-methyl-umbelliferyl ß-D-glucoside) substrate was added to 100 mL of sampled water, sealed in the Quanti-Tray, and incubated at 41 °C for 24 h, as per manufacturer instructions [66]. Following incubation, fluorescing (i.e., positive for *E. coli* and enterococci) wells were quantified using a UV light and converted, with a 95% confidence interval, into a concentration of *E. coli* and enterococci (CFU per 100 mL) using the Quanti-Tray MPN table. The *E. coli* and enterococci concentration range generated using the Quanti-Tray/MPN table system is <1 to 2419.6 CFU [66,67]. For data analyses, test results that were <1 were converted to a zero (*n* = 37), and results > 2419.6 (*n* = 25) were left as 2419.6 [6,21,37,38,39].

### 2.3. Data Analysis

Descriptive statistics were estimated for *E. coli* and enterococci concentrations for the study period. Statistical analyses were conducted using OriginLab Origin Pro 2020 (OriginLab Corporation, Northampton, MA, USA). Normality testing was completed using the Anderson Darling Test [68]. Using the 2018 National Agriculture Imagery Program (NAIP) land use and land cover data, three primary (lumped) categories of land use practices were identified. Those included mixed development, agriculture, and forested land use practices [6,21]. The land use category of mixed development included roads, impervious surfaces, mixed development, and barren areas. The agricultural assignation included lumped low vegetation, hay pasture and cultivated crops. The forested land use category included abandoned mine grass, and forested land including mixed mesophytic, dry mesic oak, and dry oak (pine) forest and small stream riparian habitats. Spearman correlation tests, a non-parametric measure of monotonic relationship between two variables, were applied to statistically evaluate the relationship between *E. coli* and enterococci concentrations, pH, and land use practices at all 22 sites, with a significance threshold of α = 0.05 [69], as per methods described in [6,21,37,38,39],. Spearman tests were also used to assess correlation between *E. coli* and enterococci concentrations and hydroclimate measured at site #13. Hydroclimate metrics included daily average stage (mm), precipitation (mm), air temperature (°C), and stream temperature (°C), daily total precipitation (mm/day), 7-day moving average of stage, and 7-day moving total precipitation [60]. Considering potentially compounding influences of several environmental variables on instream counts of fecal indicator organisms, relationships between concentrations of *E. coli*, enterococci, pH, and land use characteristics were quantified using Principal Component Analysis (PCA) [70]. Data were prone to unequal variances in the current work; therefore, scores were standardized using z-transformation. Missing data points were excluded listwise, and biplots were generated to display PCA results in a comparable way to correlation biplots (as opposed to Euclidean distance). OriginPro does not scale loadings, since scores are standardized, and loadings and scores are provided separate axes in biplots [60]. Therefore, to determine whether environmental variables were significantly (α = 0.05) contributing to observed differences in fecal indicator bacteria concentrations between sites, Canonical Correspondence Analysis (CCA) was used [71,72]. CCA is an ordination technique that utilizes environmental gradients (i.e., land use characteristics and pH) to interpret extracted axes of variation in response variables (i.e., fecal indicator data). CCA was performed using PC-ORD 7.08 software (MjM Software Design, Gleneden Beach, OR, USA).

## 3. Results

### 3.1. Climate during Study

**Figure 2:**
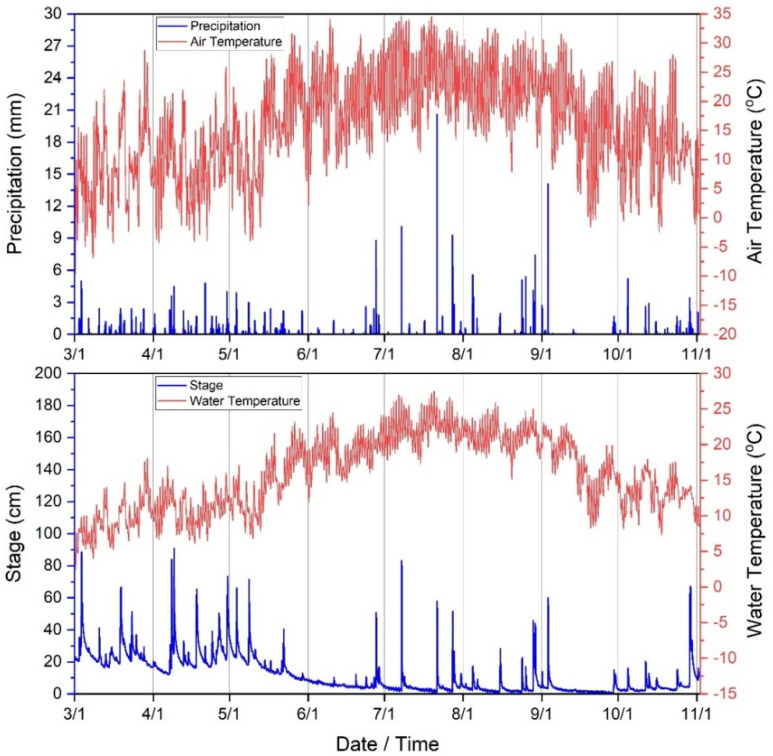
Climate variables (30−min time series) collected during the current study period (3 March−3 November 2020) in West Run Watershed, WV, USA.

Total precipitation during the period of the current study (3 March–3 November 2020) was approximately 639 mm. For context, annual average precipitation in the WRW is 1096 mm/year, dating back to 2007 [73]. The largest rainstorm occurred on 21 July at approximately 17:00 h (20.6 mm). Average stream stage (depth) was approximately 11 cm during the study with maximum stage occurring on 9 April at 04:00 h. Minimum, median and standard deviation of stage (cm) was 0.2, 5.9, and 11.35 cm, respectively during the study period. Stream water temperature (°C) mean, minimum, maximum, median and standard deviation was approximately 15.9, 2.5, 27.5, 16.0 and 5.17 °C, respectively. Air temperature (°C) mean, minimum, maximum, median and standard deviation was approximately 15.9, −9.58, 34.7, 16.2, and 8.5 (°C), respectively (Figure 2). Climate was therefore predictably variable during the study period and consistent as identified in previous publications [21,37,57,58,59,60].

### 3.2. Escherichia Coli and Enterococci Concentrations

**Figure 3:**
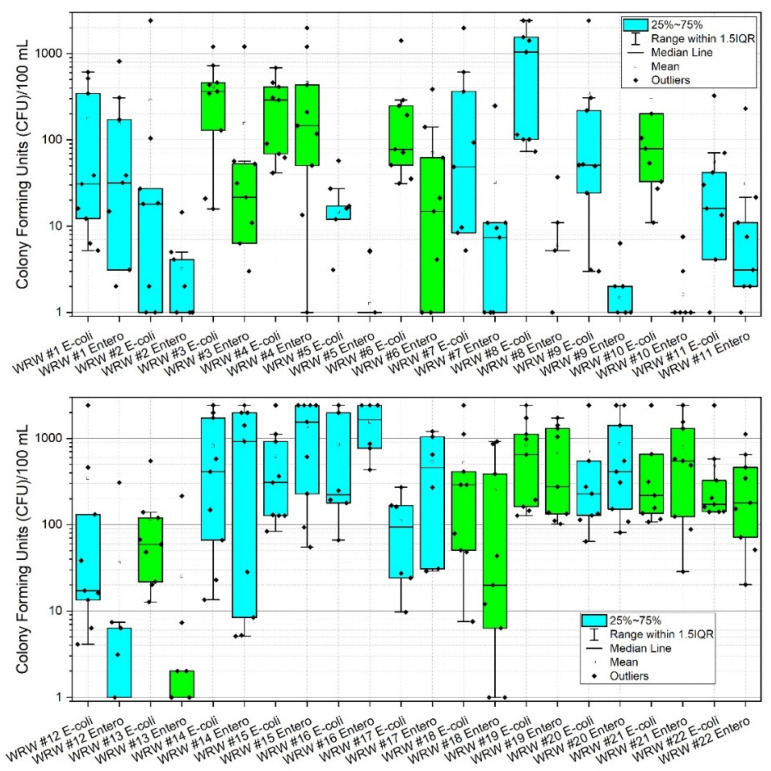
Box and whisker plots of *E. coli* and enterococci (CFU) at 22 stream monitoring sites during the study period (3 March–3 November 2020) in West Run Watershed, Morgantown, WV, USA. Colony Forming Units (CFU) are as per 100 mL water sample. Blue shaded boxes denote tributaries. Green shading denotes main stem West Run Creek. Log-scale is solely for purpose of improved visualization. Note: to reduce y-axis column heading label length, enterococci are abbreviated “Entero”.

During the study period, the highest mean concentrations (CFU) of *E. coli* were observed at sites 8, 14, 16 and 19 with values of 1027.0, 820.0, 848.9 and 836.6 CFU, respectively. Corresponding median values were 1046.2, 410.6, 221.2 and 648.8 CFU, respectively. Highest mean concentrations of enterococci (CFU) were observed at sites 4, 14, 15, 16, 20, and 21 with corresponding values of 462.7, 974.8, 1357.9, 1555.1, 873.5, and 792.1, respectively. Corresponding median values were 146.7, 920.8, 1553.1, 1643.0, 410.6 and 547.5 CFU, respectively (Appendix A, Table A1, Figure 3). Figure 3 shows box and whisker plots of all data points, where points (dots) in the graphs denote individual data points, including outliers. The open box denotes the mean and horizontal line in each plot denotes the median (see the figure legend), and the lower and upper bounds of each “box” denotes the 25% (first quartile of the data, i.e., 25% of the data lies between minimum and 25%) and 75% (third quartile of the data, i.e., 75% of the data lies between minimum and 75%) percentiles, respectively. The difference between the third quartile (75%), and the first quartile (25%) is called the interquartile range (IQR). Thus 1.5IQR is equivalent to 1.5× the IQR, where 1.5 is a scaling mechanism that controls the sensitivity of the range. Figure 3 shows a pattern of increased data variability near the approximate mid-point of the watershed (i.e., site #13), where stream reaches are responding to pulses of *E. coli* and enterococci, thus resulting in a greater range of data points. This trend returns to low and constrained values at site #17, which is a forested drainage, and site #18, where concentrations are presumably diluted by fluxes of stream water characterized by relatively low *E. coli* and enterococci concentrations emanating from the forested reach. A similar pattern is observed at sites #20 and #21, except reversed in that enterococci is of higher variability and concentrations relative to *E. coli*. Reach #20 drains a densely urbanized area of Morgantown, containing many pets and urban wildlife, thus potentially explaining these results [16,19,74,75]. Site #22 bacterial concentrations are likely diluted by contributions of the Monongahela River (e.g., back-watering). Of additional note are lower sustained concentrations of *E. coli* and enterococci at sites #1 through #13. Lower CFU’s are correlated to low pH in those reaches, consequent to historic acid-mine-drainage processes. Site #8 is an anomaly in this group for *E. coli*. The difference between *E. coli* and enterococci at site #8 in this instance is largely unknown. However, site #8 does drain a large sheep farm, thus serving presumably as a source for *E. coli* in this instance. Future research including bacterial source tracking could clarify these distinctions [13,19,20].

### 3.3. pH Results

**Figure 4:**
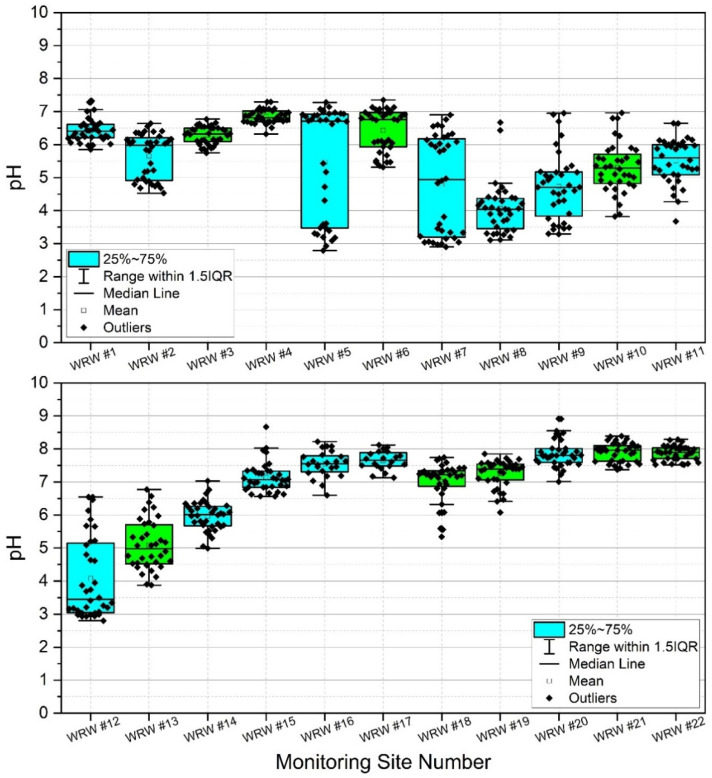
Box and whisker plots reflecting pH sampled weekly at 22 stream monitoring sites during the study period (3 March–3 November 2020) in West Run Watershed, Morgantown, WV, USA. Blue shaded boxes denote tributaries. Green shading denotes main stem West Run Creek.

Results indicated high spatiotemporal variability of pH in WRW (Appendix A, Table A2, Figure 4). Lowest mean pH was observed at sites #8 and #12, both with calculated mean pH of 4.1. However, minimum pH was observed at sites #5 and #12 (pH = 2.8). Sites #20, #21, and #22 showed the highest mean pH (pH = 7.9) during the study. Generally, lower pH was observed in the upper portions of the watershed, and higher pH was observed in the lower portion of the watershed. Given the results of previous work [6,21,38,60], it is probable that observations of chronic and acute low pH in the upper watershed are due to acid mine drainage from historic coal mining.

Surface water pH has been shown to affect bacterial colonies in freshwater lakes [76] and streams [76,77]. Given that pH is greatly affected by acid mine drainage [78,79,80] it was measured in the current work, thereby serving as a proxy for acid mine drainage and a potential explanatory variable for observed bacterial colony concentrations. Figure 4 shows box and whisker plots of pH including all data points, where points (dots) in the graphs denote individual data points, including outliers. For an explanation of the mechanics of these plots, please see Section 3.2. During the current work, the headwater reaches of the watershed (i.e., sites #1, #3, and #4) remained by majority neutral regarding pH while sites #2, #5, and #7 fluctuated from relatively low (i.e., more acidic) to neutral pH (Appendix A, Table A2). Site #8–#13 drain historic (abandoned) coal mining operations and are therefore more prone to acid mine drainage (AMD) and acidic (lower pH) conditions than many other reaches, shown clearly by the box and whisker plots. The lower half of West Run Watershed lacks former coal mining operations and therefore local AMD. However, Figure 4 shows the effect of dilution as West Run Creek becomes larger with stream distance, thereby returning low headwaters pH to nearly neutral conditions by sites #14 and #15. Site #22 illustrates this even more clearly with highly constrained pH values around neutral, likely resulting from dilution by the Monongahela River. It is noteworthy that lower pH values, and thus higher acidity, shown in Figure 4 correspond to lower CFU’s depicted in Appendix A, Table A1 and Figure 3. These helpful boxplot illustrations would not be possible without the current unique study design, including the use of many nested sites, and in situ combined *E. coli* and enterococci CFU analyses.

### 3.4. Multivariate Statistical Results

Spearman correlation tests were used to determine statistically significant (*p* < 0.05) relationships between instream fecal indicator bacteria concentrations, land use/land cover (LULC) characteristics, pH, and hydroclimate metrics (Appendix A, Table A3). Results indicated significant (*p* < 0.05) positive relationships between site-level averages, medians, and minimums of *E. coli* and enterococci. The only LULC category identified as significantly correlated with fecal indicator bacteria concentrations was mixed development, which indicated a significant (*p* < 0.05) positive correlation with *E. coli* minimum. However, results indicated significant (*p* < 0.05) positive correlations between site average pH and every enterococcus metric (i.e., site-level average, median, standard deviation, maximum, and minimum), in addition to *E. coli* minimum. Thus, correlation results emphasize the importance of surface water pH to enterococci concentrations. Hydroclimate metrics showed inconsistent relationships with fecal indicator bacteria concentrations. For example, although results indicated significant (*p* < 0.05) positive correlations between daily average stream temperature and enterococci concentration at site #5, results showed significant (*p* < 0.05) negative correlations between daily average stream temperature and enterococci concentration at site #8. Similarly, results indicated significant (*p* < 0.05) positive correlations between 7-day average stream temperature and *E. coli* concentration at site #5, but significant (*p* < 0.05) negative correlations between daily average stream temperature and *E. coli* concentration at site #8. Inconsistent correlations between fecal indicator bacteria concentrations and hydroclimate metrics could also be evidence of contrasting hydrologic flow paths and bacterial sources.

**Figure 5:**
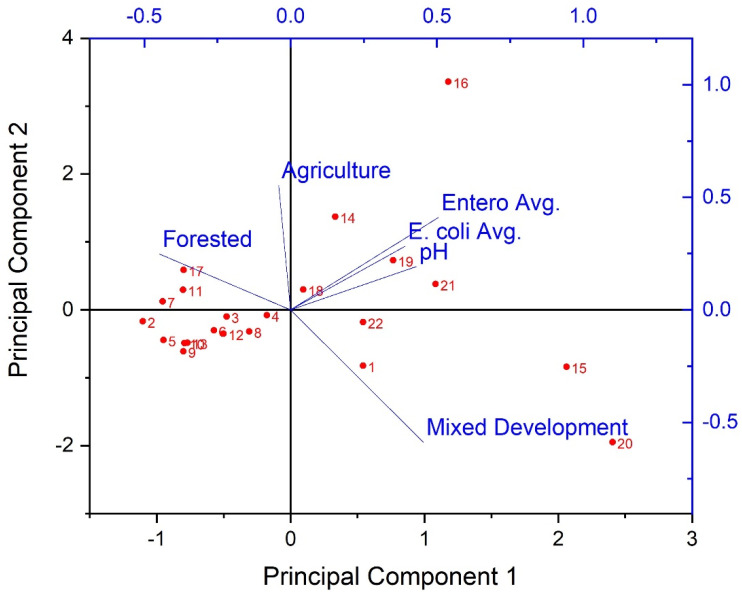
Results of Principal Components Analysis (biplot) for extracted principal components of stream fecal indicator bacteria and pH data, explaining 85% of the dataset cumulative variance during the study period (3 March−3 November 2020) and land use/land cover characteristics in West Run Watershed, WV, USA. Red labels denote study site #s; axis scale has been adjusted to maximize visibility.

**Figure 6:**
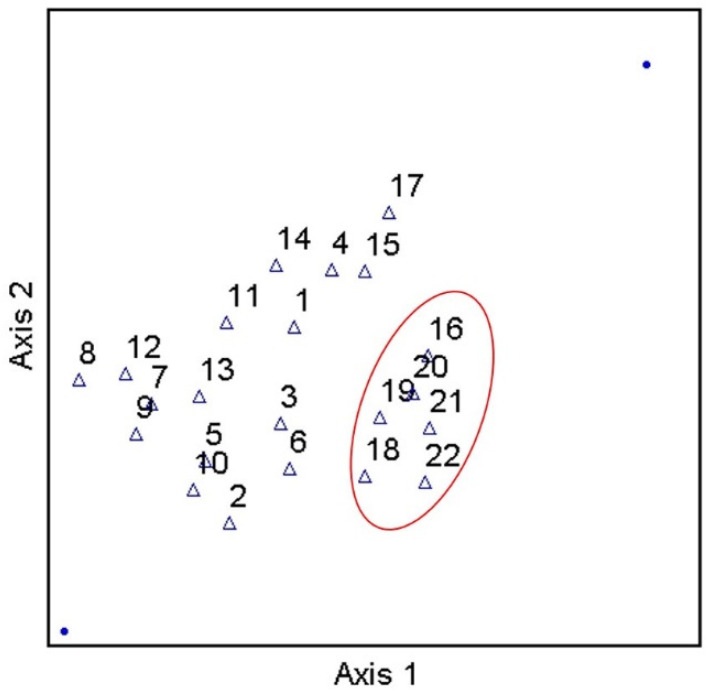
Results of Canonical Correspondence Analysis of stream fecal indicator bacteria and pH data, explaining approximately 53% of the adjusted cumulative variation of site-level bacterial averages, during study period (3 March–3 November 2020) and land use/land cover characteristics in West Run Watershed, WV, USA.

Considering the prevalence of covariation in multi-parameter environmental datasets, multivariate statistical analyses were applied to assess the correlation and/or covariation of all biological, physicochemical, and dominant land use types of the current work. PCA results showed three principal components (PCs) with Eigen values greater than 1 (Figure 5). Eigen values greater than 1 are an accepted threshold of relevance in PCA analyses [70]. The three principal components identified in the current analyses explained approximately 85% of the dataset cumulative variance. The biplot shown in Figure 5, shows clear spatial distribution (i.e., based on study site) of along PCs 1 and 2. Specifically, results suggest a negative relationship between forested land use and instream fecal indicator bacteria concentrations, and positive relationships between development, pH, and fecal indicator bacteria concentrations. Given the PCA results, a Canonical Correspondence Analysis was conducted to provide a more rigorous test of possible contributions of LULC and pH to observed spatial variability of fecal indicator bacteria concentrations (i.e., a statistically significant “effect”). Considering the significant correlations between pH and fecal indicator bacteria counts indicated by Spearman tests, pH was included as an explanatory variable in the secondary matrix (i.e., along with LULC percentages) for the CCA. CCA results showed a significant (*p* < 0.05) contribution of the environmental variables (i.e., LULC and pH) to the trends of fecal indicator bacteria data (Figure 6). For example, Figure 6 shows clear delineation of a subset of study sites (#s 16, 18, 19, 20, 21, and 22), where sufficiently high pH (i.e., ≥ 7) coincided with land use practices typically associated with loading of fecal indicator bacteria to receiving waters (development and agriculture). The second matrix explained approximately 53% of the adjusted cumulative variation of site-level bacterial averages. Therefore, although environmental variables were shown to contribute to the spatial variability of instream bacterial concentrations, nearly half of the variability was unexplained, indicating the contribution of other factors not captured in the current work. 

It is important to note that two considerations drove the selection of PCA and CCA as the multivariate methods used in the work. First, results of normality testing indicated an absence of normality for several variables (e.g., bacterial concentration data, pH, LULC, hydroclimate data). As such, the nonparametric tests PCA and CCA were a more appropriate choice for the work than multiple linear/logistic regression. Second, the results of Spearman correlation tests showed a lack of consistent monotonic (much less linear) relationships between bacterial data, pH, and LULC (i.e., present for some variables at some sites, but not across the range of samples/sites). Therefore, PCA and CCA were again a more appropriate choice since the methods do not solely rely on linear relationships between variables. Moreover, considering the potential for “threshold” characteristics in the relationships between instream bacterial concentrations, pH, and LULC, the selected multivariate methods comprise a better option for conducting meaningful analysis of the results.

## 4. Discussion

### 4.1. Escherichia coli and Enterococci Concentrations, pH, and Land Use Practices

In general, enterococci concentrations were highest at monitoring sites #14, #15, #16, and #20, with average values over the period of study of 975, 1358, 1555, and 874 CFU per 100 mL, respectively. Lowest average enterococci concentrations were observed in monitoring sites #2, #5, #8, #9, and #10, with corresponding values of approximately 3.2, 1.3, 6.0, 1.5, and 1.6 CFU per 100 mL, respectively. Monitoring sites #14, #15, #16, and #20 had dominant land use practices of forest (#14), mixed development (#15), agriculture (#16) and mixed development (#20). Importantly, the sites with lower enterococci values tended to correspond to sites with historic mining practices and acid mine drainage. These findings are supported by corresponding average pH values of 5.7, 5.5, 4.1, 4.7, and 5.3, respectively over the period of study (Appendix A, Table A2, Figure 4). Results of the various statistical tests conducted for the current work support the conclusion that pH is exerting a limiting influence on fecal indicator bacteria concentrations in WRW, specifically enterococci. Although PCA and CCA results suggest an influence of land use/land cover, fecal indicator bacteria concentrations appear subject to an inflection point co-located (i.e., mid-watershed) with an increase in pH.

**Figure 7:**
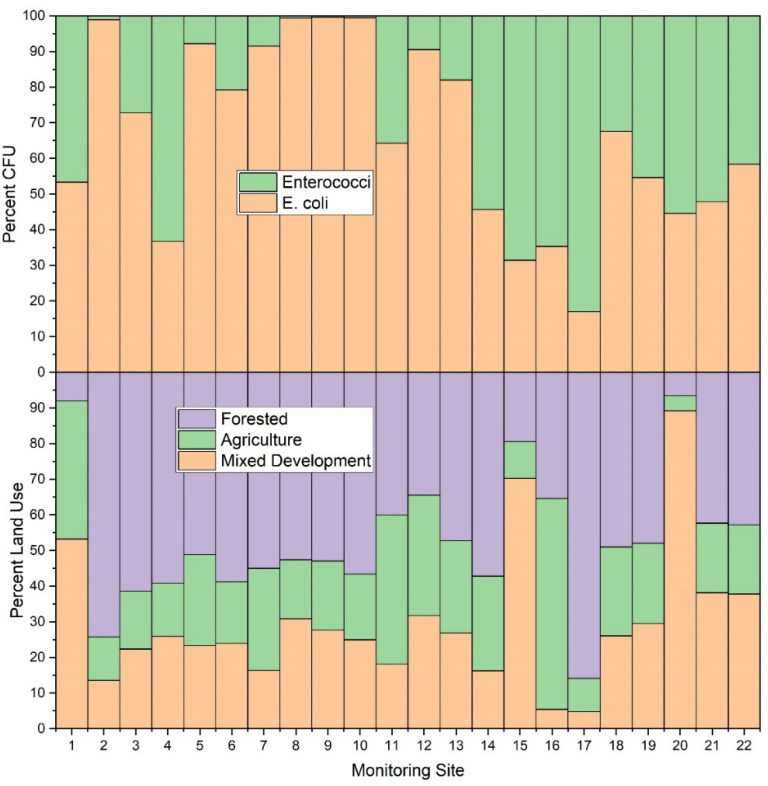
Average *E. coli* and enterococci concentration (CFU per 100 mL) and relative land use (%) for each sampling location (*n* = 22) during the study period (3 March–3 November 2020) in West Run Watershed, Morgantown, WV, USA.

**Figure 8:**
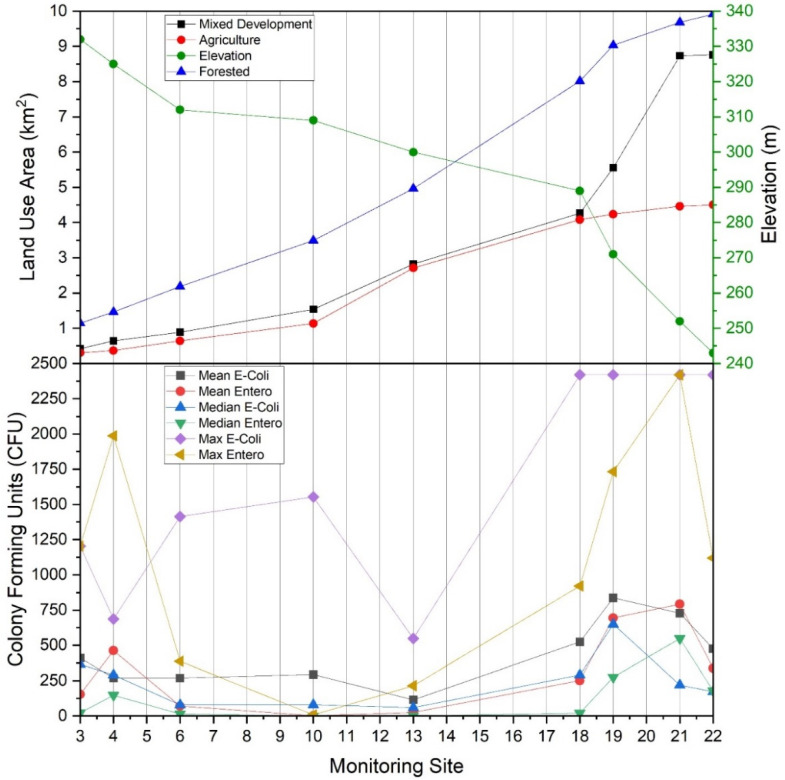
(Top) Land use type by area (km^2^) draining to main stem West Run Creek monitoring sites #3, #4, #6, #10, #13, #18, #19, #21 and #22, and elevation (m) for each monitoring site, relative to (Bottom) average (mean), median and maximum *Escherichia coli* and enterococci concentrations (CFU per 100 mL) in West Run Watershed, Morgantown, WV, USA.

In the current study, stream water samples from forested sub-catchments generally had lower *E. coli* and enterococci concentrations relative to other land use types (i.e., agricultural, and mixed development) (Figure 7 and Figure 8). This finding is in agreement with previous studies that also showed decreased fecal concentrations in forested areas [81]. Results of the current study, and previous researchers, attribute this finding to general and overall improved quality of freshwater in forested areas [82]. Despite this general agreement, there are, however, interesting nuances in the data of the current study, made possible by the number of study sites, that are worth exploration. For example, *E. coli* monitoring sites #14, #15, #16 and #17 showed similar patterns (i.e., average enterococci values higher than *E. coli* values), despite varying dominant land use practices. Dominant land use practices included forest (57%), mixed development (70%), agriculture (59%), and forest (86%) for monitoring sites #14, #15, #16 and #17, respectively. Given relative percentages of mixed development (16% and 5%, respectively) in catchments draining to monitoring sites #14 and #16, it may be assumed that the likely source of *E. coli* and enterococci at those sites was agriculture. This is a reasonable assumption since, even though agriculture was the smallest areal land use practice at monitoring site #14, the sub-catchment is approximately 3.36 km^2^ in size. Therefore, agriculture comprises approximately 0.9 km^2^, which is large relative to the total area of sub-catchments draining to monitoring sites #15 and #16 (0.98 and 0.25 km^2^, respectively). Thus, the overall contribution of agricultural practices (largely beef cattle grazing) was much greater in catchment #14, relative to #16, at the time of this study. Sub-catchment #15 (0.98 km^2^) included urban housing and shopping centers (approximate area 0.69 km^2^ of a total 0.98 km^2^), with agriculture only comprising approximately 10% of the sub-catchment, (i.e., 0.1 km^2^). It is worth noting that the limitations of the IDEXX system result in maximum value of 2419.6 CFU per 100 mL. Monitoring sites #15 and #16 surpassed the detection limit for enterococci values in those sub-catchments. It therefore is probable that actual enterococci values were higher than those detected in the current work. Monitoring site #16 is a drainage from a dairy cow facility, thus explaining the high *E. coli* and enterococci values. 

Monitoring site #20 drained an area of 3.42 km^2^ with percentage cover of forest, agriculture, and mixed development of 7%, 4 % and 89%, respectively. At the time of the current work, this sub-catchment was, by majority, urban housing (Figure 1). Large enterococci concentrations (even relative to *E. coli*) observed from this sub-catchment may be cause for concern, given there was very little agriculture and the only animal contributions of fecal coliform(s) would presumably be from small mammals (pets, urban wildlife) and humans. This may be of particular relevance considering that sewers are largely combined (i.e., human sewage and stormwater runoff) in this portion of Morgantown, WV, and system leakages or overflows during periods of high precipitation may drain to receiving waters, despite detention efforts. Thus, the risk of human exposure in sub-catchment #20 may be elevated, particularly given human population density. 

*E. coli* and enterococci concentrations generally increased from the headwater reaches of the West Run Watershed to the confluence with the Monongahela River (Figure 3 and Figure 8). In the current work, acid mine drainage (AMD) and accompanying low pH values may account for the observed lower *E. coli* and enterococci concentrations in the headwater’s regions of the watershed (as presented above). In the lower elevations of the watershed, other land use practices (e.g., non-mining practices) may be the dominant influence of *E. coli* and enterococci concentrations. For example, at West Run Creek monitoring sites #14 through #21, there were notable increases in cumulative *E. coli* and enterococci concentrations and concurrent increases in the areal extent of agricultural and mixed development land use practices. Previous researchers reported increased fecal contamination associated with increased agricultural and urban areas [74,81,83,84]. Those authors, and others, often attribute(d) those observations to increased sources (e.g., livestock manure) [22] concentrated run-off during precipitation events [74], as well as urban stream syndrome [85]. Thus, previous investigations, though lower in sampling size, or sampling locations, seem to generally support the findings of the current investigation. Importantly, the high number (*n* = 22) and land use variability of monitoring sites of the current study enable questions regarding bacterial concentrations and species distributions related to specific land use practices, and the relative flow-paths and distances of practices (e.g., development) to receiving waters. Further investigation of flow-path complexity and flow-path distances to receiving waters relative to bacterial concentrations and assemblages may provide impetus for future work. 

### 4.2. Study Implications and Future Work

It is worth acknowledging that the experimental watershed study approach [36] and high number of monitoring sites used in the current investigation facilitated collection of a distinctive data set (*n* = 396). Collectively, results highlight the importance of anthropogenic pressures to fecal indicator bacteria regimes of receiving waters. In WRW, increased instream fecal indicator bacteria concentrations were typically observed at study sites characterized by greater-than-neutral pH and either mixed development or agricultural land use types. These results have important implications. First, pH may exert a limiting influence on instream fecal indicator bacteria concentrations in WRW; and may therefore be masking the influence of LULC mediated bacteria regimes in upper portions of the watershed. This interpretation is supported by a comparison of fecal indicator bacteria concentrations at sites #12 and #16. Both catchments are characterized by livestock production (e.g., pastured cattle). However, pH was higher at site #16 than site #12, which positively correlates with higher fecal indicator bacteria concentrations at site #16. Therefore, in watersheds characterized by variable pH, investigators and managers should interpret fecal indicator bacteria concentrations carefully, to avoid overlooking a potential contamination source due to the variable masking-effect of low pH. The potential masking of LULC influences by pH was further supported by contrasting concentrations of *E. coli* and enterococci and site #8, which was characterized by the lowest average pH in the watershed. Given high *E. coli* concentrations relative to nearby sites, an active bacterial source was likely present in the vicinity of site #8. However, this spatial relationship was absent in the enterococci data, suggesting possible suppression of the land use/bacterial signal by low instream pH. Moreover, since enterococci appear to be more “sensitive” to low pH, investigators and resource managers should exercise caution when utilizing this genus of bacteria as an indicator of land use impacts on water safety. The second important implication concerns the pH regime in WRW. Although pH is presented in the current work as an independent environmental variable, alongside LULC characteristics, previous work characterized the relationship between pH and historic land use practices in the watershed [60,61]. Specifically, historic coal mining in the upper portion of the watershed resulted in continuing acid mine drainage and low pH in West Run and impacted tributaries. Although past mining activities are not fully captured/quantified in the LULC dataset utilized for the current study, given reforestation/vegetative succession of past mine sites, pH could be considered a proxy variable for such historic land use impacts and should therefore be understood to represent an integration of past and contemporary land use influences.

## 5. Conclusions

In the current investigation, principle component analysis (PCA) results indicated consistent spatial differences between bacteria concentrations, pH, and land use/land cover characteristics of study sites. Results of canonical correspondence analysis indicated a significant (*p* < 0.05) effect of sub-watershed scale land use/land cover characteristics and surface water pH on fecal indicator bacteria concentrations. Results provide evidence of the limiting effect of pH on stream fecal indicator bacteria, and the potential masking of land use influences on fecal bacteria used as water quality indicators in low pH surface waters. For example, historic coal mining in the headwaters of the study watershed resulted in acid mine drainage and acidic water (i.e., low pH) resulting in low(er) *E. coli* and enterococci colony forming units (CFUs) than might have been otherwise expected. Thus, results emphasize the importance of historic land use impacts (legacy effects) on contemporary water quality regimes and support careful consideration of past practices by land and water resource managers. 

## Figures and Tables

**Table 1:** Land use/land cover characteristics (% cover) and total drainage area (km^2^) of 22 monitoring sites (and associated sub-catchments) in West Run Watershed (WRW), West Virginia, USA. Land use percentages may not sum to 100%, as not every category is included (e.g., wetland, open water). Some categories are combinations of others (e.g., mixed development = urban + residential). Final row (Site #22) indicates the total values for the entire watershed. Shading denotes dominant land use practice by site.

Site	Mixed Development (%)	Agriculture (%)	Forested (%)	Drainage Area (km²)
1 *	53.23	38.70	8.07	0.30
2 *	13.58	12.20	74.21	0.29
3	22.35	16.17	61.32	1.87
4	25.88	14.91	59.00	2.48
5 *	23.35	25.51	51.14	0.38
6	23.91	17.25	58.70	3.72
7 *	16.33	28.60	54.91	0.78
8 *	30.78	16.47	52.35	1.55
9 *	27.57	19.33	52.84	2.29
10	24.92	18.40	56.49	6.18
11 *	18.15	41.87	40.00	1.75
12 *	31.77	33.72	34.51	1.75
13	26.83	25.77	47.15	10.53
14 *	16.19	26.43	56.92	3.36
15 *	70.28	10.31	19.42	0.98
16 *	5.38	58.72	35.16	0.25
17 *	4.78	9.38	85.84	0.75
18	25.98	24.88	48.86	16.41
19	29.45	22.45	47.85	18.88
20 *	89.16	4.19	6.61	3.42
21	38.10	19.46	42.23	22.93
22	37.71	19.38	42.66	23.24

* first and second order confluence tributaries.

## Data Availability

The datasets generated during and/or analyzed during the current study are available from the corresponding author on reasonable request.

## Appendix A

**Table A1:** Descriptive statistics of *E. coli* and enterococci concentrations (CFU per 100 mL) at each sampling location (*n* = 22) during the current study period (3 March–3 November 2020) in West Run Watershed, WV, USA. Shaded columns identify tributaries to main stem West Run Creek (unshaded).

	***E-coli*—Site Number**										
	Site #1	Site #2	Site #3	Site #4	Site #5	Site #6	Site #7	Site #8	Site #9	Site #10	Site #11
Mean	176	288	411	269	15	268	348	1027	348	294	56
Median	31	18	365	291	12	78	49	1046	51	79	16
Minimum	5	0	16	41	0	31	0	73	3	11	0
Maximum	613	2420	1203	687	57	1414	1986	2420	2420	1553	326
Std Dev	232	754	354	211	18	415	613	929	739	475	98
	***E-coli*—Site Number**										
	Site #12	Site #13	Site #14	Site #15	Site #16	Site #17	Site #18	Site #19	Site #20	Site #21	Site #22
Mean	345	115	820	622	849	110	524	837	703	727	476
Median	17	59	411	308	221	94	291	649	228	219	172
Minimum	4	13	14	84	66	10	8	127	64	108	141
Maximum	2420	548	2420	2420	2420	272	2420	2420	2420	2420	2420
Std Dev	746	158	900	727	967	97	744	767	927	919	700
	**Enterococci—Site Number**										
	Site #1	Site #2	Site #3	Site #4	Site #5	Site #6	Site #7	Site #8	Site #9	Site #10	Site #11
Mean	154	3	154	463	1	70	32	6	1	2	31
Median	32	1	22	147	0	15	7	0	1	1	3
Minimum	0	0	0	1	0	0	0	0	0	0	0
Maximum	816	15	1203	1986	5	387	249	37	6	8	231
Std Dev	254	4	372	644	2	120	77	11	2	2	71
	**Enterococci—Site Number**										
	Site #12	Site #13	Site #14	Site #15	Site #16	Site #17	Site #18	Site #19	Site #20	Site #21	Site #22
Mean	36	25	975	1358	1555	538	251	695	873	792	339
Median	1	1	921	1553	1643	459	20	276	411	548	179
Minimum	0	0	5	55	435	29	1	102	81	29	20
Maximum	308	214	2420	2420	2420	1203	921	1733	2420	2420	1120
Std Dev	96	67	945	1037	874	466	363	630	910	761	339

**Table A2:** Descriptive statistics of pH by sampling location (*n* = 22) during the study period (3 March–3 November 2020) in West Run Watershed, WV, USA. Shaded cells identify tributaries to main stem West Run Creek (unshaded). pH values reflect weekly sampling during the study period.

	**Site Number**										
	Site #1	Site #2	Site #3	Site #4	Site #5	Site #6	Site #7	Site #8	Site #9	Site #10	Site #11
Mean	6.4	5.7	6.3	6.8	5.5	6.4	4.8	4.1	4.7	5.3	5.5
Median	6.4	6.0	6.3	6.8	6.7	6.8	4.9	4.0	4.7	5.3	5.6
Minimum	5.9	4.5	5.8	6.3	2.8	5.3	2.9	3.1	3.3	3.8	3.7
Maximum	7.3	6.6	6.8	7.3	7.3	7.4	6.9	6.7	7.0	7.0	6.7
Std Dev	0.3	0.7	0.3	0.2	1.7	0.6	1.5	0.8	1.0	0.7	0.7
	**Site Number**										
	Site #12	Site #13	Site #14	Site #15	Site #16	Site #17	Site #18	Site #19	Site #20	Site #21	Site #22
Mean	4.1	5.1	6.0	7.1	7.5	7.6	7.0	7.3	7.9	7.9	7.9
Median	3.5	5.0	6.0	7.1	7.6	7.7	7.2	7.4	7.8	8.0	7.9
Minimum	2.8	3.9	5.0	6.6	6.6	7.1	5.3	6.1	7.0	7.4	7.5
Maximum	6.5	6.8	7.0	8.7	8.2	8.1	7.7	7.9	8.9	8.4	8.3
Std Dev	1.2	0.8	0.4	0.4	0.4	0.3	0.6	0.4	0.4	0.3	0.2

**Table A3:** Results of Spearman correlation tests of *E. coli* and enterococci results, pH, and land use/land cover metrics for each sampling location (*n* = 22) during the study period (3 March–3 November 2020) in West Run Watershed, Morgantown, WV, USA.

		*E. coli* Avg.	*E. coli* Med.	*E. coli* Std. Dev.	*E. coli* Max.	*E. coli* Min.	Entero Avg.	Entero Med.	Entero Std. Dev.	Entero Max.	Entero Min.	pH	Mixed Dev.	Ag	Forest
*E. coli* Avg.	SCC	1													
*E. coli* Avg.	*p*-value	--													
*E. coli* Med.	SCC	0.761	1												
*E. coli* Med.	*p*-value	0.000	--												
*E. coli* Std. Dev.	SCC	0.893	0.498	1											
*E. coli* Std. Dev.	*p*-value	0.000	0.018	--											
*E. coli* Max.	SCC	0.836	0.446	0.913	1										
*E. coli* Max.	*p*-value	0.000	0.038	0.000	--										
*E. coli* Min.	SCC	0.659	0.761	0.460	0.436	1									
*E. coli* Min.	*p*-value	0.001	0.000	0.031	0.042	--									
Entero Avg.	SCC	0.540	0.594	0.408	0.356	0.648	1								
Entero Avg.	*p*-value	0.009	0.004	0.059	0.104	0.001	--								
Entero Med.	SCC	0.450	0.514	0.305	0.271	0.586	0.964	1							
Entero Med.	*p*-value	0.036	0.014	0.167	0.222	0.004	0.000	--							
Entero Std. Dev.	SCC	0.513	0.632	0.357	0.308	0.627	0.981	0.936	1						
Entero Std. Dev.	*p*-value	0.015	0.002	0.102	0.164	0.002	0.000	0.000	--						
Entero Max.	SCC	0.539	0.624	0.393	0.332	0.660	0.982	0.945	0.992	1					
Entero Max.	*p*-value	0.010	0.002	0.071	0.131	0.001	0.000	0.000	0.000	--					
Entero Min.	SCC	0.558	0.570	0.453	0.417	0.671	0.880	0.861	0.827	0.832	1				
Entero Min.	*p*-value	0.007	0.006	0.034	0.053	0.001	0.000	0.000	0.000	0.000	--				
pH	SCC	0.285	0.374	0.202	0.180	0.606	0.801	0.834	0.764	0.790	0.833	1			
pH	*p*-value	0.198	0.086	0.368	0.423	0.003	0.000	0.000	0.000	0.000	0.000	--			
Mixed Dev.	SCC	0.270	0.176	0.240	0.354	0.452	0.155	0.060	0.162	0.173	0.148	0.189	1		
Mixed Dev.	*p*-value	0.223	0.433	0.282	0.106	0.035	0.490	0.792	0.471	0.441	0.511	0.399	--		
Ag	SCC	−0.055	−0.373	−0.037	−0.089	−0.303	−0.075	−0.090	−0.153	−0.135	−0.189	−0.240	−0.149	1	
Ag	*p*-value	0.809	0.088	0.871	0.694	0.171	0.740	0.689	0.497	0.550	0.399	0.282	0.510	--	
Forest	SCC	−0.231	0.092	−0.260	−0.267	−0.229	−0.312	−0.224	−0.254	−0.262	−0.289	−0.254	−0.673	−0.391	1
Forest	*p*-value	0.301	0.684	0.242	0.230	0.305	0.157	0.317	0.255	0.239	0.192	0.255	0.001	0.072	--
